# Spray-Induced Gene Silencing (SIGS) of Dual-Target Genes (*AaGH10* and *AaSOD*) Enhances Control of *Alternaria alternata* White Leaf Spot in Chinese Chive

**DOI:** 10.3390/jof12090656

**Published:** 2026-09-02

**Authors:** Lianzhe Wang, Kehao Huang, Yixian Gou, Yutao Zhu, Mei Zhao, Chunli Liao, Huamin Zhang, Peifang Ma, Zhen Wang, Tao Zhu, Taotao Li

**Affiliations:** 1 Pingdingshan Health Food Collaborative Innovation Center, School of Life Science and Engineering, Henan University of Urban Construction, Pingdingshan 467044, China; 2 Pingdingshan Academy of Agricultural Sciences, Pingdingshan 467044, China

**Keywords:** Chinese chive (*Allium tuberosum*), *Alternaria alternata*, spray-induced gene silencing (SIGS), double-stranded RNA (dsRNA), *AaGH10*, *AaSOD*, virulence factors

## Abstract

Chinese chive (*Allium tuberosum*) suffers severe yield and quality losses from white leaf spot disease caused by *Alternaria alternata*. Spray-induced gene silencing (SIGS) presents a sustainable alternative to traditional chemical fungicides. To maximize the biocontrol efficacy of this approach, we designed dsRNAs targeting two candidate virulence-associated genes of *Alternaria alternata*: *AaGH10*, encoding a cell wall-degrading enzyme critical for host penetration, and *AaSOD*, an antioxidant enzyme crucial for reactive oxygen species (ROS) scavenging. We comprehensively evaluated the antifungal efficacy by assessing mycelial growth inhibition, spore germination, and lesion development through in vitro and in vivo assays. The results demonstrated that both single and combinatorial treatments effectively inhibited fungal growth and spore germination, thereby reducing disease incidence on detached leaves and intact greenhouse plants. The application of the dual-target dsRNA formulation achieved an 86.7% control efficacy on detached leaves. Furthermore, it significantly alleviated in vivo disease severity, decreasing the average number of necrotic lesions from 15.6 to 1.3 per leaf. These results demonstrate that the dual-target combination exerts an enhanced protective effect compared with individual interventions. This specific dual-target dsRNA formulation establishes a robust foundation for the control of Chinese chive white leaf spot disease.

## 1. Introduction

Chinese chive (*Allium tuberosum*) is a commercially important vegetable farmed all over the world. However, its production is endangered by white leaf spot disease produced by Alternaria species, which are damaging necrotrophic fungi [1]. Members of the genus *Alternaria*, including *A. alternata*, act as aggressive pathogens causing severe leaf spot diseases across diverse crops and economic losses worldwide [2,3]. The present management of this disease is mostly based on the use of chemical fungicides, which may lead to environmental contamination, pesticide residues, and resistance in pathogenic fungi [4]. Hence, the emergence of environment-friendly and sustainable biopesticides is necessary for green agriculture.

RNA interference (RNAi) is well recognized as a highly targeted technique to control severe plant diseases. Based on this natural mechanism, spray-induced gene silencing (SIGS) has been developed as an effective tool for crop protection [5,6]. SIGS uses the ability of fungi to take up RNA from the environment to silence key survival genes by direct spraying of double-stranded RNA (dsRNA) [7,8]. Although SIGS has been extensively investigated for controlling major pathogens like *Fusarium* and *Botrytis* [9,10], RNAi-based interventions against *Alternaria* species have recently emerged as a viable crop protection strategy. For instance, the application of specific dsRNAs targeting three essential genes (*Alt-a1*, *TEF1a*, and *β-Tub*) has been shown to effectively protect tomato plants against early blight [11]. Similarly, hairpin RNA-mediated silencing of the *ACTT2* gene effectively abolished ACT-toxin biosynthesis, leading to a loss of pathogenicity in the tangerine *Alternaria* brown spot pathogen [12]. Given the proven efficacy of SIGS against fungal pathogens, extending this strategy to combat *A.alternata* on Chinese chive represents a promising opportunity.

Successful colonisation of plant tissues by necrotrophic fungi, such as *Alternaria*, relies on complex pathogenic mechanisms. These processes primarily involve the secretion of cell wall-degrading enzymes to breach host physical barriers, alongside the detoxification of host-derived reactive oxygen species (ROS) to evade plant immune responses [13,14]. During the early stages of infection, necrotrophic fungi deploy an arsenal of plant cell wall-degrading enzymes (CWDEs), including glycoside hydrolases (GHs), which are indispensable for host penetration and nutrient acquisition [15]. Silencing specific glycoside hydrolase genes—such as *RsEG146* via SIGS in *Rhizoctonia solani* [16] and *FsEG1* via HIGS in *Fusarium sacchari* [17]—has proven effective in attenuating disease severity. Concurrently, pathogens must overcome host-derived reactive oxygen species (ROS) bursts; thus, ROS-scavenging enzymes such as superoxide dismutases (SODs) play a pivotal role in the oxidative stress tolerance and pathogenicity of *A. alternata* [18]. SOD acts as a critical virulence factor in this defense mechanism, making it an attractive target for RNAi-based interventions [19]. Although multi-target dsRNA has been established as a highly effective strategy for enhancing pathogen suppression [20], the rational selection of multi-targets during the early stages of infection remains challenging. In this context, we hypothesized that concurrently disrupting the pathogen’s offensive machinery (physical penetration via *AaGH10*) and defensive capabilities (oxidative stress tolerance via *AaSOD*) would enhance pathogen suppression. Accordingly, this study investigated whether this mechanistically integrated dual-target dsRNA strategy could yield superior prophylactic efficacy against *A. alternata* on Chinese chive compared with single-target dsRNA interventions. In this study, we report the targeted silencing of two candidate virulence-associated genes, *AaGH10* and *AaSOD*, in *A. alternata*. To systematically evaluate the antifungal efficacy of single- and dual-target dsRNA interference interventions via SIGS, we analyzed target gene transcript levels, mycelial growth inhibition in vitro, and assessed disease progression by measuring leaf lesion areas and lesion numbers following exogenous dsRNA application in plants. The results demonstrated that the dual-target dsRNA formulation significantly suppressed disease severity, exhibiting an enhanced control efficacy compared to single-target dsRNA treatments.

## 2. Materials and Methods

### 2.1. Fungal Pathogen and Plant Inoculation

The pathogenic fungus *Alternaria alternata* utilized in this study was originally isolated from diseased Chinese chive (*Allium tuberosum*) leaves collected in Pingdingshan (Henan Province, China). The isolate was previously identified based on morphological characteristics and molecular analysis of ITS sequencing. The resulting ITS sequence has been deposited in the NCBI GenBank database under the accession number PZ861160. This strain is preserved in our laboratory. The isolates were grown on potato dextrose agar (PDA) at 25 °C for 10 days. Chinese chive (*Allium tuberosum* cultivar ‘791’) plants were cultivated in a greenhouse at 25 °C with a 16 h light/8 h dark photoperiod and 60–70% relative humidity.

### 2.2. Transcriptome Sequencing and Bioinformatics Analysis

To identify candidate virulence factors expressed during the infection process, *A. alternata* was sampled from inoculated Chinese chive leaves at 0, 1, and 3 days post-inoculation (dpi). The 0 dpi sample was obtained by harvesting the fungal mycelia directly from the PDA medium prior to inoculation. Total RNA extraction, library preparation, Illumina sequencing, and the subsequent basic bioinformatics analysis were performed by Biomarker Technologies Corporation (Beijing, China). The high-quality clean reads were mapped to the *A. alternata* reference genome using the corresponding genome annotation files. The raw RNA sequencing data generated in this study had been deposited in the National Center for Biotechnology Information (NCBI) Sequence Read Archive (SRA) database under the BioProject accession number PRJNA1516182. Fungal differentially expressed genes (DEGs) across the infection time points were screened using the thresholds of an adjusted p-value <0.05 and |log_2_(Fold Change)| ≥ 1. Gene Ontology (GO) enrichment analysis of the differentially expressed genes (DEGs) was performed using Fungifun3 software (https://fungifun3.hki-jena.de/ (accessed on 1 September 2025)) with the background gene set configured to the whole annotated genome of *A. alternata*. GO terms with a *p*-value < 0.05 were considered significantly enriched. Gene expression levels were determined based on log_2_-transformed FPKM values. Heat maps were created with the log2-transformed FPKM values using Sangerbox 3.0 software.

### 2.3. In Vitro Production of dsRNAs

Target sequences of *AaGH10* and *AaSOD* were amplified from cDNA of *A. alternata* using particular primers, which contained the T7 RNA polymerase promoter sequence at both ends. dsRNAs were synthesized in vitro using the MEGAscript T7 Transcription Kit (Thermo Fisher Scientific, Waltham, MA, USA) according to the manufacturer’s protocol. A dsRNA to the *GFP* gene was synthesized as a negative control in addition to the target dsRNA. All primers were verified for specificity using Oligo 6.0 and BLAST 2.17.0, and potential off-target effects were predicted using siFi21 v1.2.3-0008 (https://sourceforge.net/projects/sifi21/ (accessed on 1 November 2025). All the primers are listed in Supplementary Table S1.

### 2.4. In Vitro Antifungal Bioassay and Spore Germination Tests

To examine the inhibition of mycelial growth, 5 mm agar plugs colonized with active *A. alternata* mycelia were removed and inoculated onto the centre of PDA plates, and then 100 ng/μL (30 μL) of the respective dsRNA was immediately applied directly onto the plates for each treatment [20,21]. To evaluate combinatorial efficacy, dsRNA dosage was kept consistent across all treatment groups. Specifically, single-target treatments received 30 μL of either dsAaGH10 or dsAaSOD. For the dual-target treatment, a mixture comprising 15 μL of dsAaGH10 and 15 μL of dsAaSOD was applied, preserving both total volume (30 μL) and final dsRNA concentration. Plates treated with sterile water or dsGFP were used as controls. Colony diameters were measured after being cultured at 25 °C for 8 days [11]. A total of 5 treatments were evaluated: (i) water (Ctrl); (ii) dsRNA targeting the GFP (dsGFP); (iii) dsRNA targeting the AaGH10 gene (dsAaGH10); (iv) dsRNA targeting the AaSOD gene (dsAaSOD); and (v) dual-target dsRNA (dsAaGH10 + dsAaSOD). The mycelial growth inhibition rate (%) was calculated using the formula: $\mathrm{Inhibition}\;\mathrm{rate}\;(\%)=\frac{D_{c}-D_{t}}{D_{c}-5}\times 100\%$, where $D_{c}$ and $D_{t}$ represent the average colony diameters (mm) of the control and treatment groups, respectively.

For spore germination experiments, fungal conidia were harvested and suspended in sterile water at a concentration of 1 × 10^5^ spores/mL. Spore suspensions were combined with the relevant dsRNA formulations to achieve a final dsRNA concentration of 50 ng/μL in a total reaction volume. They were incubated in a dark, humid environment at 25 °C for 48 h. The germination rate (%) was microscopically assessed by analyzing at least 100 spores per treatment [22]. All experiments were performed with at least three independent biological replicates.

### 2.5. RNA Isolation and Quantitative Real-Time PCR (qRT-PCR)

To analyze the expression profiles of target genes, *A. alternata* were collected from inoculated Chinese chive leaves at 0, 12, 24, 48, and 72 h post-inoculation (hpi). To analyze the silencing efficiency of the target genes, an in vitro liquid co-culture assay was performed as previously described [20]. Briefly, the *A. alternata* conidial suspension was uniformly mixed with the specific dsRNA formulations to achieve a final dsRNA concentration of 50 ng/μL. To rigorously control the dosage for synergy evaluation, the dual-target formulation was prepared as a 1:1 mixture of dsAaGH10 and dsAaSOD, ensuring that the total combined dsRNA concentration remained at 50 ng/μL. The fungal tissues were collected after being cultured at 25 °C for 48 h co-incubation. Total RNA was isolated and reverse transcribed to cDNA with the RNAprep Pure Plant Kit and FastKing cDNA Kit (Tiangen, Beijing, China). Gene expression levels were estimated by qRT-PCR assays, which were performed using gene-specific primers on a CFX real time PCR machine (Bio-Rad, Hercules, CA, USA). The qRT-PCR primers’ specificity and efficiency were confirmed through melting curve analysis and agarose gel electrophoresis, with each primer pair showing a PCR efficiency of 90–110%. Relative transcript levels of *AaGH10* and *AaSOD* were normalized to the fungal internal reference gene *AaActin*. Relative gene expression levels were determined using the 2^−ΔΔCt^ method [23]. The expression level of the water-treated control (Ctrl) group was set to a reference value of 1.0. Transcript levels of all other treatment groups were calculated and expressed as fold changes relative to the Ctrl group. All the primers were listed in Supplementary Table S1.

### 2.6. Detached Leaf Assays and Synergistic Interactions Analysis

To evaluate prophylactic efficacy, in vitro detached leaf assays were performed with slight modifications based on previously established SIGS protocols [20,21]. Briefly, healthy, fully expanded Chinese chive leaves were excised, surface-sterilized, and uniformly sprayed with the respective dsRNA formulations at a concentration of 50 ng/µL. At 24 h post-treatment, each leaf was drop-inoculated with 20 μL of an *A. alternata* spore suspension (1 × 10^5^ spores/ mL) and incubated in a high-humidity room. To minimize developmental variance, fully expanded leaves at the same developmental stage were harvested from the same node position of different individual plants. Necrotic lesion areas (cm^2^) were determined at 3 dpi. All experiments were performed with at least three independent biological replicates.

The in vitro colony inhibition and in planta detached leaf control efficacy data of the dsRNA treatment were used to identify the nature of the interaction between the co-application of the two dsRNAs by application of Colby’s mathematical model [24]. The expected control efficacy (E) of the dual-target dsRNA formulation was calculated using the equation $E=X+Y-\frac{XY}{100}$, where X and Y represent the observed efficacy (%) of the individual dsRNA (% of single dsAaGH10 and dsAaSOD treatments, respectively) treatments. The synergy factor (SF) was subsequently determined as the ratio of the observed combined efficacy (O) to the expected efficacy (E). The interaction was classified as synergistic if SF >1.0, additive if SF =1.0, and antagonistic if SF <1.0.

### 2.7. Greenhouse Pot Tests in Planta

To evaluate the in vivo prophylactic efficacy of the dsRNA formulations, whole-plant spray assays were conducted based on previously established SIGS protocols with modifications [20,21]. Chinese chive (*Allium tuberosum*) plants grown in greenhouse pots were sprayed with the respective dsRNA formulations at a concentration of 50 ng/μL until the leaves were fully wetted but prior to runoff. The dsRNA solutions (and the water control) were supplemented with 0.01% (*v*/*v*) Tween-20. After 24 h treatment, all plants were inoculated by spraying an *A. alternata* spore suspension of $1\times 10^{5}$ spores/mL [25]. For reliable quantification of disease severity, a total of 30 leaves (10 uniform leaves per pot across three independent biological replicates) were sampled from each treatment group at 7 dpi. Disease severity was evaluated by quantifying the average number of distinct necrotic lesions per leaf.

### 2.8. Statistical Analysis

The data, presented as mean ± standard error (SE), were obtained from at least three independent experiments. To satisfy parametric assumptions, discrete lesion counts were subjected to a square-root transformation prior to analysis. The data were then subjected to a one-way analysis of variance (ANOVA) followed by Tukey’s pairwise comparison test (*p* < 0.05).

## 3. Results

### 3.1. Identification and Expression of the Candidate Target Genes

**Figure 1:**
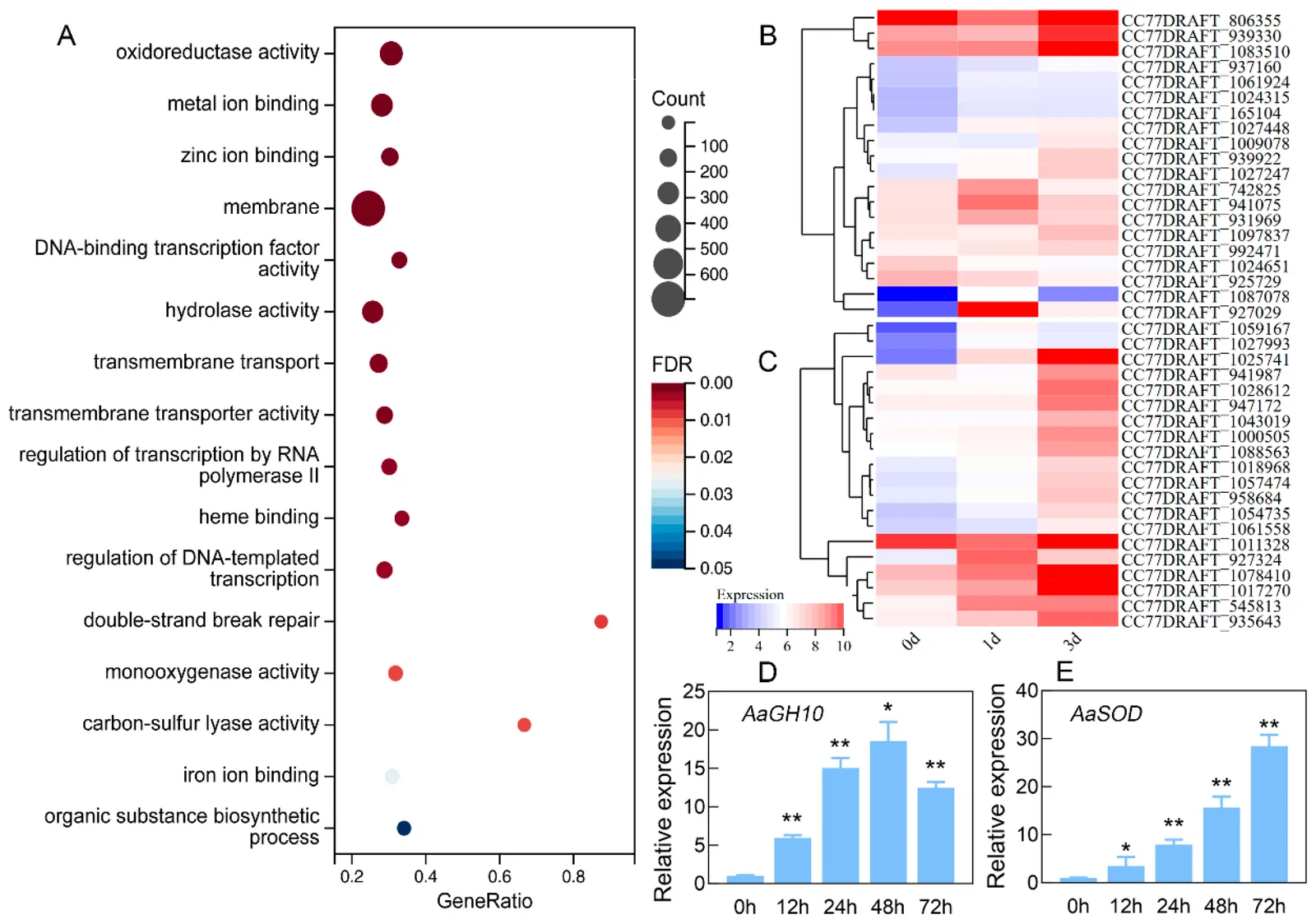
Transcriptome-based identification and expression analysis of candidate target genes in *A.alternata*. (**A**) Gene Ontology (GO) functional enrichment analysis of the differentially expressed genes (DEGs) identified in *A. alternata*. GeneRatio represents the proportion of DEGs assigned to a specific enriched term relative to the total number of annotated DEGs in the input list. (**B**,**C**) Heatmap representation of DEGs expression associated with hydrolase activity (**B**) and oxidoreductase activity (**C**) based on log_2_-transformed FPKM values. (**D**,**E**) qRT-PCR validation of the expression patterns of the candidate target gene *AaGH10* (**D**) and *AaSOD* (**E**). Data are presented as the mean ± standard deviation (SD) of three independent biological replicates. Significant differences between the post-inoculation and control are indicated as * *p* < 0.05; ** *p* < 0.01.

To choose appropriate RNAi target genes for the control of Chinese chive white spot disease caused by *A. alternata*, comparative transcriptome sequencing of *A. alternata* was performed in an infection time course at 0, 1 and 3 days post-inoculation (dpi). Gene Ontology (GO) enrichment analysis of the differentially expressed genes (DEGs) indicated a specific activation of pathogenesis-related biological activities. Interestingly, several important functional modules were considerably enriched in GO analysis, including oxidoreductase activity, metal ion binding, and hydrolase activity (Figure 1A, Supplementary Table S2).

To screen the candidate virulence factors, the top 20 elevated genes based on oxidoreductase or hydrolase activities were extracted and displayed in a heatmap (Figure 1B,C, Supplementary Table S3). Based on the transcriptomic profiles, with expression levels quantified as $\log_{2}\left(\mathrm{FPKM}\right)$ values, two candidate target genes exhibited distinct and significant upregulation patterns during the infection process. Specifically, the target gene CC77DRAFT_927029, which encodes a glycoside hydrolase family 10 (GH10) protein and associated with hydrolase activity, showed a low expression level of 1.6 at 0 dpi. However, its expression was upregulated upon infection, peaking at 10.5 at 1 dpi and declining to 6.2 at 3 dpi. Conversely, the target gene CC77DRAFT_1025741, which encodes a superoxide dismutase (SOD) and related to oxidoreductase activity, maintained a low baseline expression of 1.8 at 0 dpi, but demonstrated up-regulation to 6.9 at 1 dpi and 10.4 at 3 dpi. Compared with other potential candidates, these two genes maintained low expression levels prior to infection but were robustly induced (exceeding an expression value of 10) at 1 or 3 dpi. Given their remarkable up-regulation characteristics during pathogenesis, these two genes were selected as target genes for further functional characterization.

To validate the transcriptomic data, the expression profiles of the two target genes (*AaGH10* and *AaSOD*) were assessed by qRT-PCR at 0, 12, 24, 48, and 72 h post-inoculation (hpi). Consistent with the RNA-seq results, both genes were significantly upregulated during the infection time course. Specifically, the relative expression of *AaGH10* peaked at 48 hpi, exhibiting an 18.5-fold upregulation. In contrast, *AaSOD* demonstrated a continuous upregulation post-inoculation, and the relative expression reached 28.4-fold at 72 hpi (Figure 1D,E).

### 3.2. Antifungal Activity of the Multi-Target dsRNAs In Vitro

**Figure 2:**
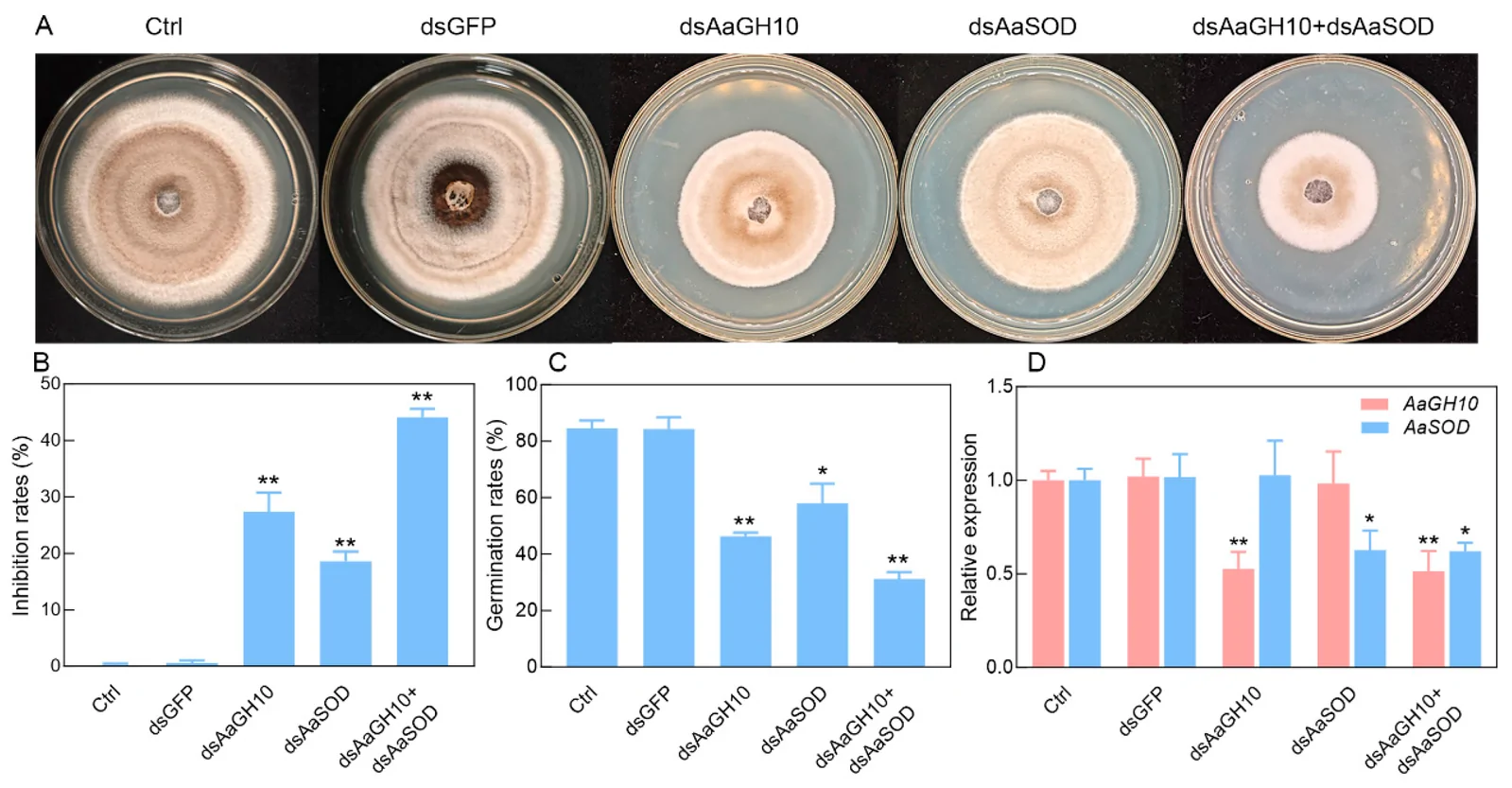
In vitro antifungal activity and gene silencing efficiency of dsRNA treatments against *A. alternata*. (**A**) Phenotypic images of *A. alternata* colony growth on PDA plates under the different treatments. *A. alternata* were treated with sterile water (Ctrl), dsGFP, single-target dsRNAs (dsAaGH10 or dsAaSOD), and the dual-target dsRNA (dsAaGH10 + dsAaSOD). (**B**) Quantitative analysis of the colony growth inhibition rates derived from the colony diameter measurements. (**C**) Spore germination rates of the different treatment groups. (**D**) Relative transcript levels of the target genes *AaGH10* and *AaSOD* in *A. alternata* following different treatments by qRT-PCR to confirm specific gene silencing efficacy. Data are presented as the mean ± standard deviation (SD) of three independent biological replicates. Significant differences between the post-inoculation and control are indicated as * *p* < 0.05; ** *p* < 0.01.

Following the production of specific dsRNAs, we first evaluated their target specificity in silico. The result confirmed that the designed dsRNAs exhibit specificity, with no perfect 21-nt contiguous matches identified against the transcriptomes of the Chinese chive host (*Allium tuberosum*), humans (*Homo sapiens*), or representative beneficial insects (*Apis mellifera*), suggesting a low potential for off-target effects in the organisms analyzed. Then, we examined the direct antifungal potential of the designed dsRNAs (Figure 2A,B, Supplementary Table S4). The application of individual dsRNAs obviously inhibited the colony growth of *A. alternata*, with single inhibition rates of 27.3% and 18.6% for dsAaGH10 and dsAaSOD, respectively. Interestingly, the co-application of the two dsRNAs resulted in a significant reduction in fungal growth, with a total inhibition rate of 44.1%. The mathematical examination by Colby showed that the theoretical predicted additive inhibition was 41.1% with the corresponding Synergy Factor (SF) of 1.07 (SF > 1.0), which mathematically indicates an enhanced combinatorial effect on fungal growth suppression.

The effects of single or combination dsRNAs on the germination of *A. alternata* conidia were also determined (Figure 2C, Supplementary Table S5). The controls showed a high normal germination rate of more than 84.4% while single treatments with the individual dsRNAs substantially compromised spore viability. In the single dsAaGH10 and dsAaSOD treatment groups, germination rates were restricted to 46.3% and 58.0%, respectively. Moreover, the dual-target treatment maintained stronger suppressive pressure and reduced the absolute germination rate to 31.2%. The results showed that using two targets of dsRNA can significantly reduce spore germination rates.

To ascertain whether these phenotypic impairments were indeed driven by specific target gene silence, we subsequently quantified the transcript levels using qRT-PCR (Figure 2D, Supplementary Table S6). The results demonstrated that the endogenous transcript levels of *AaGH10* and *AaSOD* were significantly down-regulated compared with the Ctrl and dsGFP treatments. Notably, the co-treatment reduced their relative transcript levels to 0.51 and 0.62 (representing knockdown efficiencies of 49% and 38%, respectively), confirming successful RNAi-mediated silencing.

### 3.3. Protection of Chinese Chive Leaves Against Alternaria Infection in Detached Condition by Spray-Induced Gene Silencing

**Figure 3:**
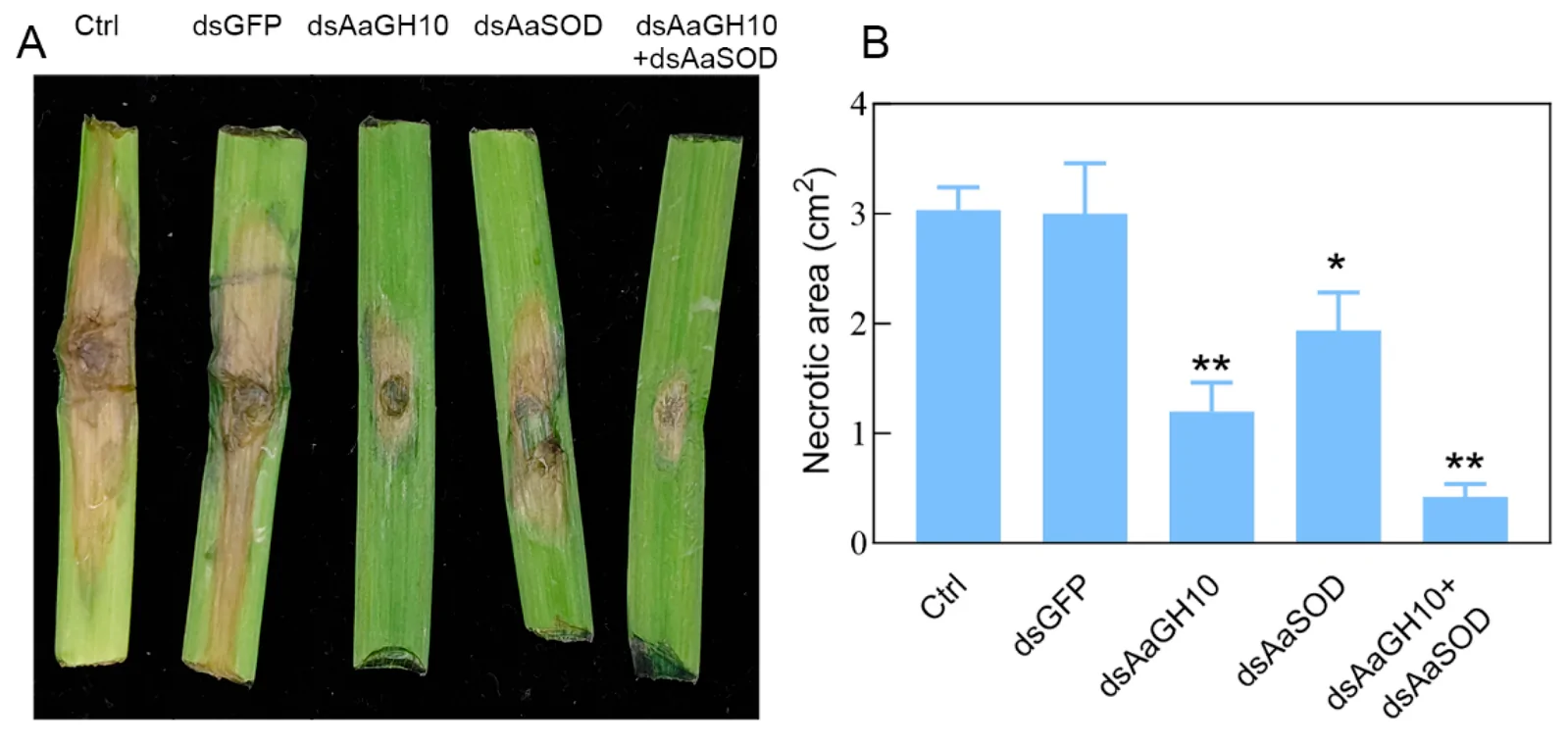
Infection symptoms of dsRNA treatments against *A. alternata* on Chinese chive leaves. (**A**) Disease phenotypes on detached Chinese chive leaves at 3 dpi. Leaves were treated with sterile water (Ctrl), GFP-dsRNA, single-target dsRNAs (dsAaGH10 or dsAaSOD), and the dual-target dsRNA (dsAaGH10 + dsAaSOD) prior to *A. alternata* inoculation. (**B**) Quantitative analysis of the lesion areas (cm^2^) across different treatment groups. Data are presented as the mean ± SD from three independent biological replicates. Significant differences between the different treatments and control are indicated as * *p* < 0.05; ** *p* < 0.01.

To assess whether the in vitro antifungal activity also translates into protection against disease in planta, we used a spray-induced gene silencing (SIGS) test on detached Chinese chive leaves. Leaves were prophylactically sprayed with specific dsRNAs and then inoculated with *A. alternata*. At the end of the evaluation, leaves treated with the control and dsGFP control developed severe symptoms, with an average lesion area of 3.0 cm^2^. In contrast, prophylactic spray of target-specific dsRNAs significantly slowed the progression of illness symptoms (Figure 3A).

Quantitative investigation of the necrotic areas confirmed the protective efficacy of the dsRNA treatment. Single applications of dsAaGH10 and dsAaSOD dramatically reduced the lesion areas to 1.2 cm^2^ and 1.9 cm^2^ with control efficacies of 60.0% and 36.7%, respectively (Figure 3B, Supplementary Table S7). In the dual-target treatment, the necrotic lesion size was limited to 0.4 cm^2^, and the total control efficacy was 86.7%. The computed anticipated efficacy was 74.7% and the synergy factor (SF) was 1.16, as predicted by Colby’s calculation. These data indicate that the combined therapy has an enhanced protective effect in planta.

### 3.4. Greenhouse Pot Assays In Vivo Verify the Protective Effect of the dsRNA

**Figure 4:**
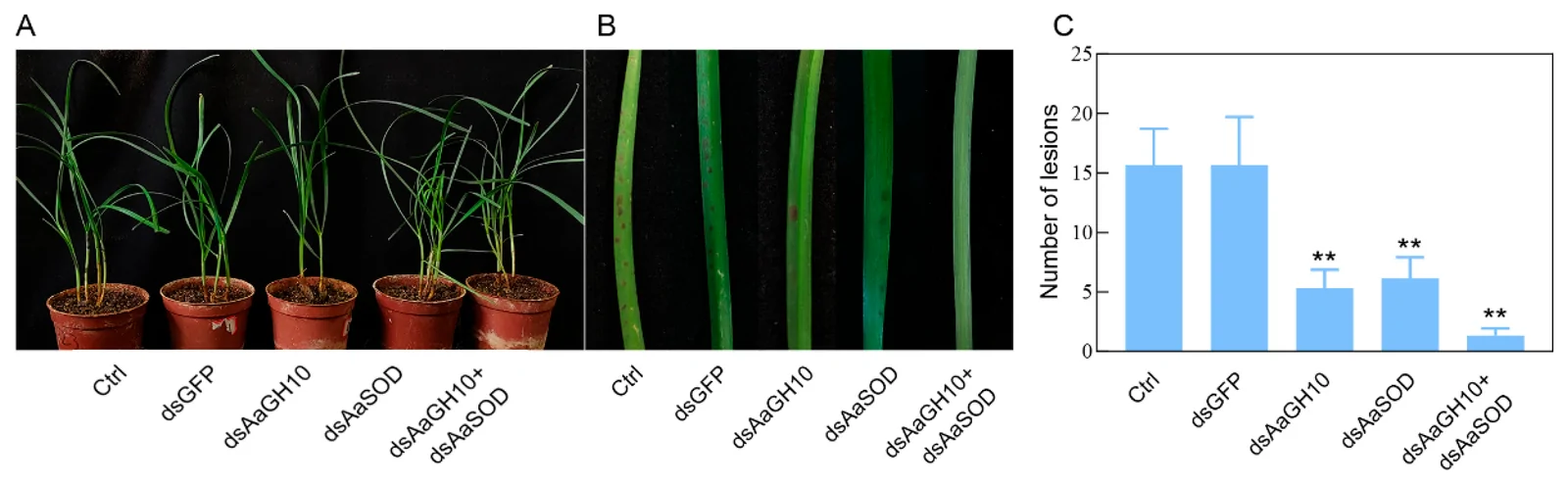
In vivo prophylactic efficacy of SIGS-mediated dsRNA treatments against *A.alternata* on potted Chinese chives. (**A**) Representative whole-plant disease phenotypes of potted Chinese chives at 7 dpi. Plants were prophylactically treated with sterile water (Ctrl), GFP-dsRNA, single-target dsRNAs (dsAaGH10 or dsAaSOD), or the dual-target dsRNA (dsAaGH10 + dsAaSOD) prior to fungal challenge. (**B**) Necrotic lesions on the infected leaves across different treatment groups. (**C**) Quantitative analysis of the average number of lesions per leaf in different treatment groups. Data are presented as the mean ± SD from three independent biological replicates. Significant differences between the different treatments and control are indicated as ** *p* < 0.01.

To examine the potential of the dsRNA method for practical application, in vivo greenhouse pot studies were undertaken with whole Chinese chive plants. At the evaluation endpoint, plants in the mock and dsGFP control groups showed extensive infection with many localised grey necrotic lesions on the leaves (Figure 4A,B). In contrast, SIGS with target-specific dsRNAs visibly reduced these illness symptoms, resulting in fewer spots on the leaves. Further, across all treatment groups, assessment revealed no observable chlorosis, necrosis, or stunting, nor any adverse effects on leaf morphology or expansion following application of any exogenous dsRNA formulation.

As infection under greenhouse circumstances was spot-like, disease severity was quantitatively measured as the average number of necrotic lesions per leaf. In the control groups, significant infection was noted with an average of 15.6 independent lesions per leaf. Single treatments of dsAaGH10 and dsAaSOD considerably lowered the frequency of infection, with the number of lesions per leaf being 5.3 and 6.2, respectively. Importantly, the dual-target dsRNA treatment gave the best protection with the disease suppressed to an average of only 1.3 lesions per leaf (Figure 4C, Supplementary Table S8). These in vivo measurements reveal that the dsRNA formulation in combination successfully inhibits fungal penetration and establishment under greenhouse conditions.

## 4. Discussion

The extensive use of traditional chemical fungicides to manage *A. alternata* caused serious environmental and resistance issues, demanding the development of eco-friendly solutions. Spray-induced gene silencing (SIGS) is a promising crop protection technique. In this study, we demonstrated the successful use of a dual-target exogenous dsRNA strategy as a target-specific and effective combinatorial approach for suppressing the phytopathogenic fungus *A. alternata* in Chinese chive. Through systematic in vitro and in planta assessments, we showed that the simultaneous application of dsRNAs targeting both *AaGH10* and *AaSOD* significantly inhibited mycelial growth and spore germination in vitro, while effectively restricting lesion expansion and reducing lesion numbers in planta. These findings provide compelling evidence that combinatorial SIGS holds great potential as an alternative to conventional chemical fungicides for disease management.

The efficacy of SIGS largely depends on the successful uptake of environmental dsRNA by the target pathogen, a capacity that varies among fungal species [8]. Consistent with a recent study where dsRNA treatments reduced *A. alternata* DNA loads in plant tissues by 7- to 27-fold [11], our results indicate down-regulation of *AaGH10* and *AaSOD* transcripts alongside phenotypic impairments. Together, these observations suggest that *A. alternata* is capable of internalizing exogenous dsRNAs to initiate the RNAi pathway. However, direct visual tracking (e.g., via fluorophore-labeled dsRNA) remains a valuable direction for future investigations.

The prophylactic efficacy observed in our study highlights the importance of target gene selection. In this study, we strategically selected *AaGH10* and *AaSOD*—both of which function as virulence factors—rather than conventional housekeeping genes. *AaGH10* acts as an offensive virulence factor necessary for physical penetration, while *AaSOD* serves as a defensive pathogenicity factor crucial for detoxifying host-derived reactive oxygen species (ROS). Our preliminary transcriptomic analysis revealed that the expression of both genes was significantly upregulated during the early stages of *A. alternata* infection, indicating their indispensable roles in establishing pathogenesis (Figure 1). Both in vitro antifungal activity assays and SIGS-mediated *in planta* lesion analyses demonstrated that individually silencing *AaGH10* and *AaSOD* in *A. alternata* yielded robust fungal growth inhibition and disease suppression (Figure 2, Figure 3 and Figure 4). Many plant cell wall-degrading enzymes (PCWDEs) are secreted by fungal pathogens to overcome the host’s physical barriers. GH10 endo-xylanases are essential for hemicellulose depolymerization [15]. To withstand the highly antagonistic host environment, the pathogen depends extensively on SOD and other ROS-scavenging enzymes to dismantle host-derived superoxide radicals [18]. *SOD* functions as a critical defensive virulence factor that mediates the detoxification of both endogenous intracellular ROS and host-derived extracellular oxidative bursts, and is indispensable for successful infection [26]. Targeting these pathogenesis-specific virulence factors directly disrupts the infection machinery, imposing a more severe fitness constraint on the pathogen compared with silencing essential housekeeping genes. Housekeeping genes are fundamental for survival, but fungi frequently possess compensatory metabolic mechanisms or duplicated genes that can partially buffer the impact of their suppression. This rationale is strongly corroborated by recent evidence demonstrating that suppressing major virulence effectors, such as the *Alt-a1* allergen, provides enhanced disease control against *A. alternata* than targeting conserved structural or metabolic genes like *TEF1a* or β-tubulin [11].

Furthermore, in single-target analyses, SIGS-mediated silencing of *AaGH10* exhibited superior prophylactic efficacy compared with silencing *AaSOD*. Specifically, treatment with dsAaGH10 resulted in a higher in vitro colony growth inhibition rate (27.3%) than dsAaSOD (18.6%) and concurrently restricted the detached leaf lesion area to 1.2 cm^2^, which was notably smaller than that observed with the dsAaSOD treatment (1.9 cm^2^) (Figure 2C and Figure 3B). This differential efficacy can be attributed to their distinct spatiotemporal roles during the infection process. *GH10* encodes a crucial cell wall-degrading enzyme essential for breaching the host’s physical barriers, whereas SOD primarily acts downstream to neutralize host-derived oxidative bursts. In a prophylactic SIGS setting, directly halting this initial penetration imposes a more immediate and severe constraint on fungal establishment than impairing subsequent stress-tolerance mechanisms. This mechanistic observation is strongly corroborated by recent RNAi studies. For instance, the application of spray-induced gene silencing (SIGS) targeting *RsEG146*, a glycoside hydrolase family 16 gene in *Rhizoctonia solani*, effectively attenuated the severity of sheath blight in maize [16]. Targeted silencing of *FsEG1*—a critical virulence factor encoding a GH12 family protein secreted by *Fusarium sacchari*—via host-induced gene silencing (HIGS) effectively confers host resistance against Pokkah Boeng disease [17].

However, relying on conventional RNAi strategies that target a single lethal gene may exert strong selective pressure, potentially driving pathogen adaptability and the evolution of resistance [27,28]. To overcome this limitation, our study employed a dual-target strategy. Most importantly, co-targeting physical penetration (*AaGH10*) and oxidative tolerance (*AaSOD*) exerted an enhanced protective effect, evidenced by a Synergy Factor (SF) of 1.16 in detached leaves (Figure 3B). While the suppression of *AaGH10* blunted the pathogen’s physical ingress, the simultaneous silencing of *AaSOD* compromised its ROS-scavenging capacity, leaving the invading hyphae highly vulnerable to host-derived oxidative stress. Collectively, our data demonstrated that this dual-target dsRNA formulation provides enhanced disease control and holds significant promise for mitigating the evolution of fungal resistance in agricultural settings.

To rigorously benchmark the commercial viability of our dual-target dsRNA strategy, we directly compared its efficacy with currently dominant chemical fungicides in agricultural practice. Historically, conventional chemical fungicides, including triazoles (e.g., difenoconazole) and strobilurins (e.g., azoxystrobin), have been widely applied due to their anti-Alternaria activities and established field control efficacy [29]. The potent baseline activity of triazoles is supported by in vitro assays demonstrating that tebuconazole achieves a high mycelial growth inhibition rate of 83.57% [30]. In contrast, benzimidazole-based agents exhibit markedly reduced efficacy: carbendazim–mancozeb mixtures yield only 63.33% inhibition, while single-agent carbendazim drops to 21.02% under identical assay conditions [30]. Recently, co-formulated products such as fluopyram·tebuconazole (35% SC) deliver field protective efficacies of 86.75–95.81% [31]. In this paper, our dual-target dsRNA formulation achieves a control efficacy of 86.7%, which is comparable to that of high-quality chemical fungicides. Furthermore, unlike chemical fungicides that drive resistance evolution and leave persistent toxic residues, dsRNA molecules are environmentally labile and highly target-specific, making this dual-target SIGS strategy an ideal solution for frequently harvested leafy vegetables like Chinese chive.

Although the dual-target dsRNA formulation demonstrated protective efficacy, this effectiveness is currently viable primarily under controlled greenhouse conditions. The transition to practical field application is fundamentally limited by the environmental instability of naked RNA molecules against ultraviolet (UV) irradiation, microbial nucleases, and rain wash-off [32,33]. Consequently, achieving sustained crop protection in the field will strictly depend on the future development and integration of stable carriers, such as nanomaterials (e.g., LDH clay nanosheets, Carbon Dots, and Carbon Nanotubes) [34,35,36,37,38]. Nevertheless, the rapid environmental degradation and strict sequence specificity of dsRNA, as corroborated by our in silico off-target evaluations, offer a favorable biosafety profile, effectively mitigating the risk of toxic chemical residues on plants [32,39]. Ultimately, while the dual-target SIGS strategy provides a theoretical foundation for RNA-based biocontrol, its practical application in agriculture remains contingent upon the advancement of these delivery technologies.

In conclusion, our results demonstrate that the dual-target dsRNA formulation against *AaGH10* and *AaSOD* overcomes the inherent limitations of single-target interventions. While widespread field applications are currently constrained by production costs and regulatory burdens, the recent emergence of commercialized RNA-based plant protection products highlights the practical feasibility of this technology. By continuing to optimize dsRNA stability and delivery vehicles, this combinatorial RNAi strategy has the potential to become a highly sustainable and eco-friendly tool for managing Chinese chive white leaf spot disease and other destructive fungal pathogens.

## 5. Conclusions

In this study, we developed a dual-target SIGS strategy against *Alternaria alternata* by simultaneously silencing *AaGH10* (offensive) and *AaSOD* (defensive). Co-application of dsRNAs significantly inhibited mycelial growth and spore germination, achieving 86.7% control efficacy on detached leaves and reducing greenhouse lesions from 15.6 to 1.3 per leaf. The synergistic effect (SF = 1.16) confirmed enhanced protection and may potentially contribute to reducing the risk of resistance development. This eco-friendly dsRNA approach provides a promising, fungicide-comparable alternative for managing Chinese chive white leaf spot.

## Data Availability

The raw RNA sequencing datasets generated and analyzed during the current study are available in the NCBI SRA repository under the BioProject accession number PRJNA1516182.

## Supplementary Materials

The following supporting information can be downloaded at https://www.mdpi.com/article/10.3390/jof12090656/s1: Table S1: The primer sequences used in the study; Table S2: Gene Ontology (GO) enrichment analysis of the differentially expressed genes (DEGs) of A.alternata; Table S3: The expression data (log2-based values) of the candidate target genes at 0, 1, and 3 dpi; Table S4: The colony growth inhibition rates of the different treatment groups; Table S5: The spore germination rates of the different treatment groups; Table S6: The relative transcript levels of the target genes AaGH10 and AaSOD in A. alternata following different treatments by qRT-PCR; Table S7: The lesion areas (cm²) across different treatment groups; Table S8: The average number of lesions per leaf in different treatment groups.
